# Extraction and structural analysis of *Angelica sinensis* polysaccharide with low molecular weight and its lipid‐lowering effect on nonalcoholic fatty liver disease

**DOI:** 10.1002/fsn3.1581

**Published:** 2020-05-18

**Authors:** Ping Ma, Congyong Sun, Wenjing Li, Wenwen Deng, Michael Adu‐Frimpong, Jiangnan Yu, Ximing Xu

**Affiliations:** ^1^ Key Lab for Drug Delivery and Tissue Regeneration Jiangsu Provincial Research Center for Medicinal Function Development of New Food Resources School of Pharmacy Jiangsu University Zhenjiang China

**Keywords:** *Angelica sinensis* polysaccharide, lipid metabolism, low molecular weight, NAFLD, optimized purification process

## Abstract

Nonalcoholic fatty liver disease (NAFLD) is one of the prevalent and typical chronic liver diseases. In this study, we extracted a novel *Angelica sinensis* polysaccharide (ASP) with low molecular weight (MW) of 3.2 kDa through optimized “one‐step” purification process. The major monosaccharide components of ASP were mannose, rhamnose, glucuronic acid, galactose, arabinose, and xylose with weight ratio of 0.23:0.17:14.41:0.39:1.68:0.87, respectively. Herein, “small” ASP could serve as an effective therapeutic option for NAFLD both in free fatty acid‐induced L02 models and in high‐fat diet‐induced mice models. Results revealed that low MW ASP dose‐dependently decreased TG, TC in vitro and TG, TC, ALT, HDL‐C, and LDL‐C in vivo. Oil Red O‐positive area and Nile red fluorescence intensity decreased in ASP treatment groups both in vitro and in vivo which suggested ASP could reduce lipid accumulation and fatty regeneration. Hematoxylin–eosin staining results shown a decrease in hepatocytes ballooning indicating that ASP could ameliorate liver lipid degeneration. Briefly, a novel polysaccharide with low MW was successfully obtained which can prospectively act as NAFLD therapy.

## Graphical abstract

ASP with low molecular weight could be obtained through easy “one‐step” purification process. The “small” and easy‐purified ASP possesses high biostability, repeated feasibility, and availability. The novel ASP can be alternative for treatment of NAFLD.

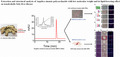

## 1 INTRODUCTION

Nonalcoholic fatty liver diseases (NAFLD) is the hepatic manifestation of the metabolic syndrome and is defined as the accumulation of fat in the absence of any secondary causes including a significant alcohol consumption, steatogenic drugs, or hereditary disorders (Buzzetti, Pinzani, & Tsochatzis, 2016). As a clinicopathological syndrome, term of NAFLD encompasses a wide spectrum of hepatic disorders, which ranged from simple steatosis to serious nonalcoholic steatohepatitis (NASH), fibrosis and cirrhosis with its clinical consequences (Ratziu, Bellentani, Cortez‐Pinto, Day, & Marchesini, 2010). NAFLD has been recognized as one of the most common chronic liver diseases in western countries. Epidemiological studies have suggested that over 30% of general population are affected by NAFLD with 4%–5% of them usually progressing to NASH (Goh & McCullough, 2016). Pathologically, this culminates in an impaired survival because of the accompanied symptoms involving cardiovascular and liver‐related diseases. However, the underlying mechanism of NAFLD is complex and multifactorial. Various advanced diagnosed skills have been developed in the past decades, namely ultrasound, computed tomography, or magnetic resonance imaging (Neuschwander‐Tetri, 2017), albeit almost no therapeutic strategy being verified to be effective apart from lifestyle modification (Hannah & Harrison, 2016; Orci et al., 2016). Current drugs utilized in clinical trials could be classified into the following kinds, viz., antioxidants, insulin sensitizers, and lipid‐lowering agents (Gossard & Lindor, 2011; Takahashi, Sugimoto, Inui, & Fukusato, 2015), which usually focus on one type of targets. Pertinently, NAFLD involves numerous steps of hepatic lipid homeostasis; thus, medicines with multitargeting ability are urgently needed.

Polysaccharides commonly exist in most traditional Chinese medicines (TCM) and encompassed a wide range of bioactivities including anti‐inflammatory, antitumor, antioxidative, hepatoprotective, and antifatigue (Xie et al., 2016; Yu, Shen, Song, & Xie, 2018). Based on these excellent values, huge efforts have been put into digging out effective herbal polysaccharides for NAFLD treatment. Currently, large progresses have been made with one major research being *Ginkgo biloba* leaf polysaccharide which is reported to play a certain protective role against high‐fat diet (HFD)‐induced NAFLD via the attenuation of insulin resistance, preservation of liver functions, elevation of antioxidant defense system (Xu, Zhang, & Wang, 2017), and reduction of fatty pre‐oxidation (Yan et al., 2015). Moreover, polysaccharides obtained from *Chicory* (Wu et al., 2018), *Caulis dendrobium* (Xu et al., 2017), *Schiandra* (Wang, Yuan, et al., 2016), *Lyciumbarbarum* (Omari‐Siaw et al., 2016; Xiao, Wang, Liong, So, & Tipoe, 2018), and *Radix Hedysari* (Sun, Wang, Duan, Shang, & Cheng, 2014) have been designated as excellent alternatives for clinical purpose. However, the ability of *A. sinensis* polysaccharide (ASP) to improve NAFLD symptoms has rarely been demonstrated. Notwithstanding, Wang, Cao, et al. (2016) reported that ASP could remarkably alleviate serum and lipid disorders as well as fatty hepatosis via the mediation of relevant signal pathway. Based on the authors' conclusion, it is possible that ASP could be applied as dietary supplement or healthcare substance to ameliorate metabolic syndrome in population that consistently consume HFDs; thus, it is imperative to investigate the improved anti‐NAFLD activity of ASP.

As TCM, *A. sinensis* (Oliv.) Diels is abundant in nature and is known for its outstanding therapeutic effects on hematological and gynecological conditions. The ASP one of the major effective components of the plant possesses various pharmacological effects and bioactivities covering antidiabetes, antitumor (Zhang et al., 2016), antifatigue, antioxidation, hepatoprotective ability, and immunostimulatory effects (Pan, Jiang, & Wu, 2018). Existing literature on the extraction and purification techniques of ASP has shown that current skills mostly focus on traditional water extraction and ethanol precipitation followed by gel filtration or ion‐exchange chromatography purification process (Jin, Zhao, Huang, Xu, & Shang, 2012), such as Sephadex G‐100 and DEAE‐Sephadex A‐25. Based on these techniques, various kinds (approximately 36) of ASP with the molecular weight (MW) ranging from 5.1 to 740 kDa have successfully been obtained (Zhang et al., 2016). However, ASP has some challenges such as fast metabolism, low stability, and decreased repeated feasibility (Gu et al., 2018, 2019) resulted from its large MW and complex purification processes, which restrict its application in the clinical setting. Thus, an easy‐repeat purification process to obtain low MW and stable ASP is urgently needed.

In the present work, ASP with low MW was isolated via an optimized “one‐step” purification process. The well‐known traditional water extraction and alcohol precipitation methodology were adopted to acquire crude polysaccharides. Then, the crude extraction was purified by D315 weak‐base ion‐exchange resin which is believed to play an effective role in purification and absorption (Fan et al., 2018). After lyophilization, ASP (MW = 3.2 kDa) was obtained and subjected to free fatty acid (FFAs)‐induced L02 cell lines in vitro. It was speculated that ASP could decrease the level of triglyceride (TG) and total cholesterol (TC) level in supernatant. Also, through Oil Red O (ORO) and Nile red staining, ASP was postulated to ameliorate lipid accumulation in FFAs‐induced cells. In order to investigate the lipid‐lowering function of ASP in vivo, NAFLD model was established by feeding the ICR male mice (3–4 weeks) with HFD.

## 2 MATERIALS AND METHODS

### 2.1 Materials

The dry roots of *A. sinensis* were purchased from Zhenjiang Zhilin Pharmacy. The monosaccharide standards (Glu, D‐Glu, Xyl, Rha, Man, Ara, Gal, Fuc, and lactose) were purchased from Aladdin Industrial Corporation while the dextran MW standards (Dextran 670,000, 270,000, 80,000, 25,000, and 5,000 Da) were bought from Sigma‐Aldrich. Congo red and other chemicals/solvents were purchased from Sinopharm Chemical Reagent Co., Ltd. Test kits for TG, TC, alanine aminotransferase (ALT), high‐density lipoprotein cholesterol (HDL‐C), low‐density lipoprotein cholesterol (LDL‐C), and bicinchoninic acid were purchased from Nanjing Jiancheng Bioengineering Institute. ORO, palmitic acid (PA), and oleic acid (OA) were purchased from Sigma‐Aldrich while Nile red was bought from Aladdin Industrial Corporation.

### 2.2 Preparation and characteristic of ASP

#### 2.2.1 Preparation of ASP

The dry roots of *A. sinensis* were bought from Zhenjiang Zhilin Pharmacy. The crude polysaccharide was obtained by hot‐water extraction and ethanol precipitation. Briefly, dry powders of *A. sinensis* (400 g) were soaked with three volumes of 85% ethanol at 80°C for 2 hr to remove lipids and pigments, followed by filtration. The resultant filtrate was decocted twice with 10 volumes of hot water at 80°C for 3 hr. All the aqueous extracts were then gathered, centrifuged, and concentrated to the desired volumes. Next, 95% ethanol was added into the concentrated solution till final concentration of ethanol reached 80% and was allowed to stand overnight at 4°C for precipitation to occur. Subsequently, the residues were washed with ethanol (three times), dissolved into double distilled water (DDW), and lyophilized via freeze‐drying to acquire crude polysaccharide. Afterward, the trichloroacetic acid (TCA) method was performed to remove protein. The deproteination was conducted several times until no protein was detected through Bradford method. After that, the remainder was collected, concentrated with vacuum rotary evaporator, and finally dialyzed (MW cutoff 3.5 kDa) against DDW. The retention was lyophilized and then purified with D315 weak‐base ion‐exchange resin. The column was eluted with DDW till no carbohydrate was examined by phenol–sulfuric acid method. The elution was collected, concentrated, lyophilized, and designated as ASP.

#### 2.2.2 Molecular weight of ASP

The average MW of ASP was determined by high performance liquid chromatography (HPLC) method on an Agilent 1260 instrument, which is equipped with TSK‐gel G4000PW (7.5 mm × 300 mm) column. The mobile phase was composed of 20 mM ammonium acetate, which flowed at a rate of 0.6 ml/min. After filtration through 0.45 μm membrane, an aliquot (20 μl) of the sample (1 mg/ml) was injected into column for each run. Dextrans with different MWs (MW, 5, 25, 80, 270, 670 kDa) were used as standards, and the calibration curve was acquired by plotting the retention time against the logarithm of their respective MW. The MW of sample was calculated via the calibration curve.

#### 2.2.3 Uronic acid content

The uronic acid content was determined by carbazole and sulfuric acid method, using glucuronic acid as standard. Briefly, glucuronic acid (10 mg) was dissolved into 100 ml DDW as stored solution. The various concentrations of glucuronic acid liquor were formed by diluting with appropriate DDW to final volume (1 L). After reacting with 0.025 M potassium tetraborate (potassium tetraborate was dispersed in sulfuric acid), the product was subjected to 0.125% carbazole–ethanol to develop a color. The absorbance was measured at 530 nm wavelength using a microplate reader (BioTeK Epoch). Sample (1 mg/ml) was performed in the parallel with the standard. The standard curve was obtained by plotting absorbance against concentration of glucuronic acid (data not shown). The relative uronic acid content was calculated by the following formula:

$$\text{uronic}\;\text{acid}\;\text{content}\;\left(\%\right)=W_{i}/W\times 100\%$$

where *W_i_* is uronic acid content of sample and *W* is the total weight of sample added into experiment. All the experiment processes were duplicated.

#### 2.2.4 Identification of triple‐helical structure

Congo Red technique is a widely recognized as the gold‐standard to identify the triple‐helical structure of polysaccharide (He, Shao, Men, & Sun, 2010; Zhang et al., 2019; Zhu, Pan, Han, Sun, & Chen, 2019) Therefore, Congo Red experiment was performed to identify whether ASP had triple‐helical structure. The DDW was employed as blank control. The concrete process was conducted according to previous report (Wang et al., 2013), with slight modification. Briefly, ASP (5 mg) was dispersed into DDW (2 ml) and added into 80 μM Congo Red (2 ml). Successively, appropriate volume of 1 M sodium hydroxide was fully mixed into the reaction system, reaching the final concentration of sodium hydroxide (0, 0.05, 0.1, 0.15, 0.2, 0.25, 0.3, 0.35, 0.4, 0.45, and 0.5 M). Subsequently, the resulting solution at respective different concentrations was scanned on an ultraviolet spectrophotometer from 200 to 800 nm wavelength. The resultant curve was obtained by plotting maximum absorption wavelength against sodium hydroxide concentration. The experiment was replicated three times.

#### 2.2.5 Monosaccharide composition analysis

Polysaccharide is a kind of macromolecular carbohydrate, which is consisted of at least 10 monosaccharides. The composed of monosaccharides is a critical element of polysaccharides, which is closely related to bioactivities of polysaccharides. The composition of ASP was analyzed by PMP‐label method as previously reported (Jin et al., 2018). In brief, the sample was hydrolyzed with trifluoroacetic acid (TFA) at 100°C for 6 hr. Then, the hydrolyzed production was reacted with PMP at 70°C for 30 min. The standards, including Glu, D‐Glu, Xyl, Rha, Man, Ara, Gal, Fuc, and lactose (internal reference), were treated in parallel with the sample. After several extractions, the aqueous phase was filtered into 0.45 μm membrane and separated by HPLC on an Agilent 1260 instrument (Agilent), which was equipped with Agilent ZORBAX ODS column (5 μm, 4.6 mm × 150 mm) plus a gradient elution program (Table 1). The A phase consisted of 0.1% methanoic acid and 50 mM ammonium acetate, while the B phase was acetonitrile and C phase was 0.1% methanoic acid. All the mobile phases were eluted in the column at a flow rate of 0.6 ml/min. The separation was recorded under 254 nm wavelength. The identifications were based on retention time with respect to standards. Quantification was carried out by integration of the chromatographic peak area.

**Table 1:** Elution gradients for the analysis of monosaccharide composition via HPLC

Time (min)	*A* (%)	*B* (%)	*C* (%)
0	80	20	0
6	80	20	0
6.5	0	20	80
20	0	20	80

#### 2.2.6 Fourier‐transform infrared (FT‐IR) spectrum

In order to obtain structural insights of polysaccharide, the FT‐IR were employed. The desired ASP was dried at 45–50°C and then mixed with dried potassium bromide. After fully grinding, the mixture was pressed to pellet and scanned in the mid‐infrared region (4,000–400 per cm).

#### 2.2.7 Nuclear magnetic resonance (NMR) spectroscopy

In order to obtain structural insights of polysaccharide, the NMR were employed. The ASP (30 mg) was dissolved into 500 μl D_2_O. Transient sonication or heating in few minutes contributed to dissolution. The C^13^ NMR and H^1^ NMR spectroscopy were recorded with a Bruker DPX‐500 spectrometer (Bruker Corporation), and the operating parameter was at 296.9 K with frequency of 400 MHz.

### 2.3 Protective effects of ASP on NAFLD in vitro

#### 2.3.1 Cytotoxicity assay

In vitro MTT assay was used to evaluated cytotoxicity of ASP. Briefly, L02 cells were seeded into 96‐well culture plate at a density of 5 × 10^4^ cells/well with complete medium (RMPI medium, 10% fetal bovine serum) and cultured at 37°C with 5% CO_2_ plus standard humidified atmosphere until reaching to 70%–80% confluence. Cells were treated with ASP in different concentration (200, 400, 600, 800, 1,000, and 1,200 μg/ml) for 24 hr. Besides, cells with no transfection were blank control, whose cell viability was considered as 100%. The relative cell viability was calculated by the equation:

$$\text{cell}\;\text{viability}\;\left(\%\right)=\frac{[\text{OD}490]\;\text{samples}}{[\text{OD}490]\;\text{control}}\times 100\%.$$

#### 2.3.2 Cell culture and assay

Immortalized human hepatic L02 cell lines were cultured in RMPI medium containing 10% FBS and incubated at 37°C in 5% CO_2_ and standard humidified incubator. The medium was refreshed every 2 days, and the cells were passaged with trypsin after reaching 80%–90% confluent. FFA was prepared by mixing palmitic acid and oleic acid in 1:2 weight ratio. The cells were then treated with 0.6 mM FFA for 24 hr to establish the NAFLD model in vitro. Afterward, ASP at various concentrations (200, 400, and 800 μg/ml) was added into the medium. After another 24 hr of incubation, the cells were analyzed.

#### 2.3.3 Biochemical analysis

The abnormality of serum lipid is a major risk factor of NAFLD. Thus, in order to investigate the effect of ASP in FFAs‐induced NAFLD in vitro, we firstly detected the lipid profile (TG and TC) in the cell supernatant. The cells were dissociated with trypsin and subjected to TG and TC assessment. The experiments were conducted via commercial kits (Jiancheng) in accordance with the manufacturer's instruction.

#### 2.3.4 Oil Red O (ORO) staining

To further research into the lipid‐lowering effect of ASP, ORO staining was employed to identify the extent of oleic acid lipid accumulation. The ORO staining was performed under the instructions of the manufacturer. Briefly, the cells were fixed with 4% paraformaldehyde. After three times washing with PBS, the culture plates of the cells were added into 60% isopropanol and allow to stand for 5 min. Freshly diluted oil red working solution was applied to cells for 15 min. After rinsing with 60% isopropanol, the cells were counterstaining with hematoxylin. Next, the pictures were captured with a light microscope (Nikon Ti‐E). Semiquantitative analysis of ORO‐positive area was conducted by ImageJ software.

#### 2.3.5 Nile red staining

In order to further investigate the lipid‐lowering effect of ASP, Nile red staining was applied to examine the neutral fat deposition. Nile red staining was performed according to the specifications of the manufacturer. In short, the cells were fixed with 4% paraformaldehyde for 30 min at room temperature. After thrice washing with PBS, the cells were treated with fresh Nile red working solution for 30 min. After three times rinsing with PBS, the cells were counterstained with DAPI for 30 min. Finally, the cells were imaged under a fluorescence microscope (Nikon Ti‐E).

### 2.4 Protective effects of ASP on NAFLD in vivo

#### 2.4.1 Acute oral toxicity study in mice

ICR male mice (3–4 weeks) were purchased from the animal center of Jiangsu University. The animals were grown and fed in specific light‐controlled condition (12 hr light/dark cycle) with constant temperature (23 ± 2°C) and humidity (55 ± 15%). Besides, the mice were given unrestricted access to food and water. After the mice were acclimatized for 2 weeks, they were randomly divided into two groups (*n* = 5). The normal control group received 0.5% CMC‐Na, and the ASP‐treated group received 2,000 mg/kg body weight. After a single oral administration, the whole mice were closely observed 4 hr every day for 14 days. At the 15th day (after 24 hr starvation), all mice were sacrificed and internal organs were collected for histological analysis. After fixed with 4% paraformaldehyde, internal organs were stained with hematoxylin–eosin (HE). Results were imaged under an upright microscope (Nikon Ti‐E).

#### 2.4.2 Animals and experimental designs

Mice were acclimatized for 2 weeks and 45 days of HFD feeding. Then, they were randomly divided into five groups (*n* = 5). These included model control group (the animals were given 0.5% sodium salt of carboxymethyl cellulose sodium (CMC‐Na) by gavage at a dose of 0.4 ml/20 g for 10 consecutive days) and positive control group (they were given fenofibrate by gavage in a dose of 0.4 ml/20 g for constant 10 days). Additionally, ASP high‐dose, middle‐dose, and low‐dose groups were given in dosages of 500, 300, and 100 mg/kg, respectively. The normal control group was administered with regular chow and given 0.5% CMC‐Na in dosage of 0.4 ml/20 g in the last 10 consecutive days. All experiment protocols were carried out according to regulations and guidelines of Ethic Committee of Jiangsu University (license number: 201935650).

#### 2.4.3 Biochemical analysis

Blood samples were collected at the scarifying day. TG and TC, serum ALT, HDL‐C, and LDL‐C were analyzed by commercial kits (Jiancheng) according to the specifications of the manufacturer.

#### 2.4.4 Histopathology analysis

In order to investigate the lipid accumulation in the hepatic tissues, paraformaldehyde‐fixed liver tissues were placed into optimal cutting temperature (OCT) compound and then were fractioned into 8 µm section for subsequent staining with HE, ORO, and Nile Red. Liver samples were dissected for histological assessment. OCT was adopted in this experiment. Part of frozen sections of OCT embedded samples were stained by HE. The residual sections were stained by ORO and Nile red, respectively. The histological sections stained with HE and ORO were visualized and imaged under an upright microscope (Nikon) while the Nile red staining was visualized by fluorescence microscope (Nikon Ti‐E). Semiquantitative analysis of ORO‐positive area was conducted by ImageJ software.

### 2.5 Statistical analysis

The entire data were presented as mean ± standard deviation (*SD*). Differences between the distinct groups were statistically evaluated using ANOVA and the least‐significant difference test. And *p* < .05 was considered statistically significant. The entire calculations were performed via statistical software (SPSS version 19.0, SPSS Inc.). Additionally, all the graphs were drawn using Origin Software^®^ (OriginLab Corp.). Semiquantitative analysis was conducted by ImageJ 1.44p (Wayne Rasband, National Institutes of Health).

## 3 RESULTS AND DISCUSSIONS

### 3.1 Preparation and characteristic of ASP

#### 3.1.1 Preparation of ASP

**Figure 1:**
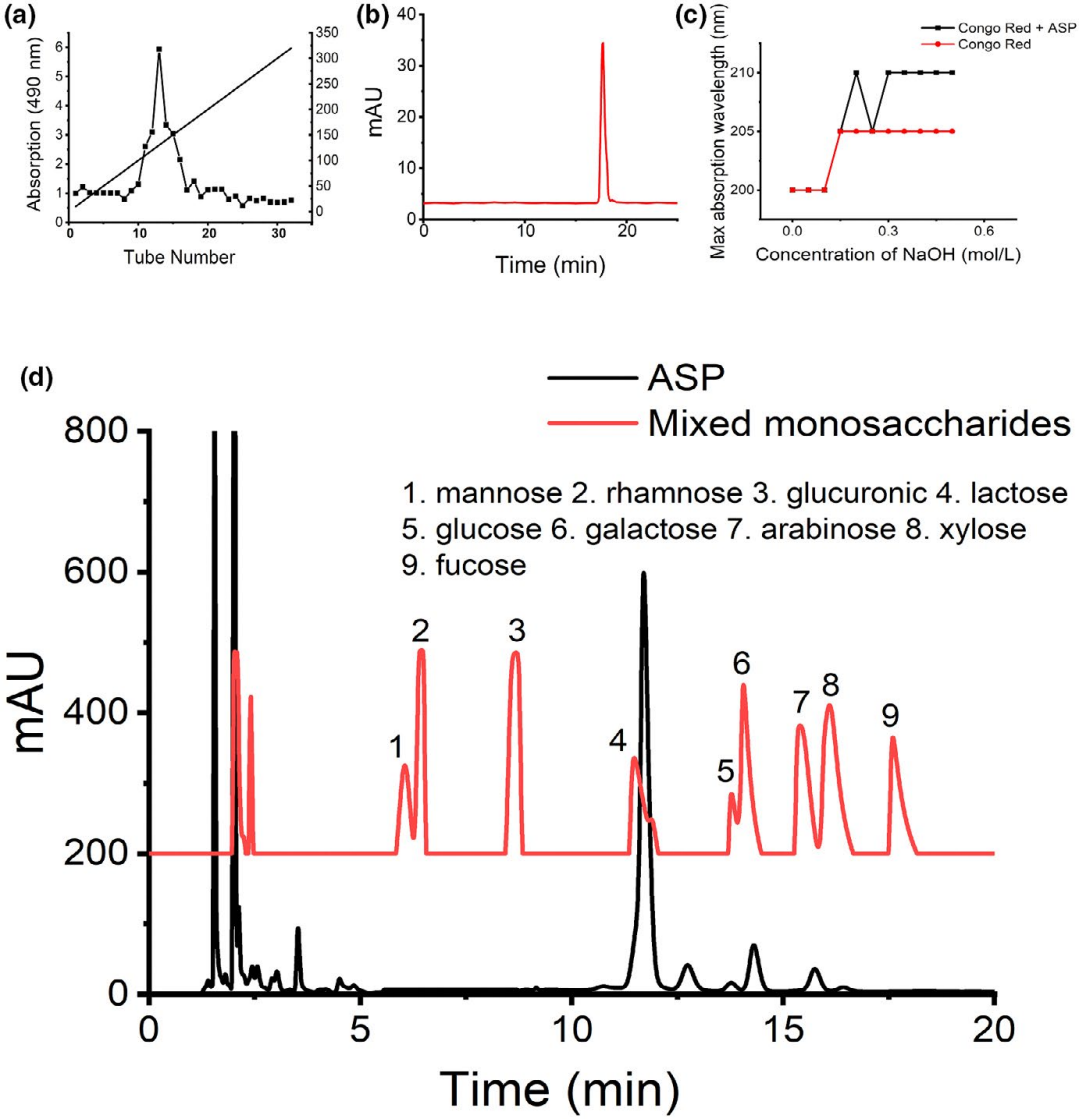
Extraction and structural analysis of *Angelica sinensis* polysaccharide. (a) Elution profile of ASP fraction on D315 column. (b) MW of ASP determined by gel chromatography, the retention time was 17.2 min while the MW was determined to be 3.2 kDa. (c) Resultant curve of Congo red experiment. Red shift could be observed suggesting that ASP possessed triple‐helical structure. (d) Monosaccharides compositions of ASP. The major monosaccharide components of ASP were mannose, rhamnose, glucuronic acid, galactose, arabinose, and xylose with weight ratio of 0.23:0.17:14.41:0.39:1.68:0.87

The crude polysaccharide was obtained by traditional water extraction and ethanol precipitation method, and the yield was nearly 10% (*w*/*w*). After removing protein with TCA, the remaining protein content was not up to 10% (*w*/*w*), which indicating that ASP was a protein bounded polysaccharide extraction and the floating protein was almost removed after TCA method. The crude polysaccharide was then purified by D315 weak‐base ion‐exchange resin, and ASP fraction could be obtained from DDW (Figure 1a). The ASP fraction was a single symmetrical narrow peak (Figure 1a), which suggested that it is homogeneous distribution. A body of studies reported that MW played a critical role in bioactivity of polysaccharides. Polysaccharides with high MW are usually in poor water solubility which hinder them exert bioactivity. The finding of HPLC showed the average MW of ASP is 3.2 kDa (Figure 1b), which is lower than other polysaccharides extracted from *A. sinensis* reported before. Thus, ASP with low MW obtained by our strategy promise an excellent solubility and activity. Study suggested that polysaccharides with high MW would be unfavorable for penetrating multiple cell membrane barrier into organism to exert pharmacological effects (Alban & Franz, 2000). Additionally, these polysaccharides with high MW are also limited by unideal injection or other dosage form resulted low water solubility and high intrinsic viscosity for clinical application (Li et al., 2016). However, It has been reported previously (Zhang et al., 2016) that polysaccharide of *A. sinensis* should be purified thrice in columns to acquire low MW ASP (5.1 kDa), which could be used to set the various uncontrolled factors for subsequent experiments. Thus, ASP with low MW (3.2 kDa) extracted through an easy‐repeat purification process in our strategy promise repeated feasibility and excellent bioactivity. The uronic acid content was determined to be 72.49 ± 0.032% using carbazole and sulfuric acid method. It is well‐known that uronic acid content could alter physicochemical property and modify solubility of polysaccharides. Polysaccharide fractions rich in uronic acid indicated high bioactivity. In present study, we extracted a novel polysaccharide with low MW from the dried roots of *A. sinensis* by optimized “one‐step” purified method, which promise ASP possessed excellent water solubility, appropriate intrinsic viscosity, and thus high bioactivity.

#### 3.1.2 Identification of triple‐helical structure

Congo Red, a kind of acid dyes, can complex with matter possessing triple‐helical structure. The maximum absorption wavelength of the formed complex could be increase as the concentration of sodium hydroxide ranges from 0 to 0.5 M. As shown in Figure 1c, the maximum absorption wavelength of the sample displayed slightly red shift in comparison with the Congo Red, indicating that ASP had triple‐helical structure.

#### 3.1.3 Monosaccharide composition analysis

The major monosaccharide components of ASP were mannose, rhamnose, glucuronic acid, galactose, arabinose, and xylose with weight ratios of 0.23:0.17:14.41:0.39:1.68:0.87, respectively (Figure 1d). Notably, monosaccharides such as mannose, rhamnose, galactose, arabinose, and xylose had been identified to have intensive effect on anti‐inflammatory (Yang et al., 2019). Galactose was designated as “brain sugar” for its strong functions on cell formation, immune system operation and promoting anti‐inflammatory responses (Brockhausen, 2006).

#### 3.1.4 Fourier‐transform infrared (FT‐IR) spectrum and nuclear magnetic resonance (NMR) spectroscopy

**Figure 2:**
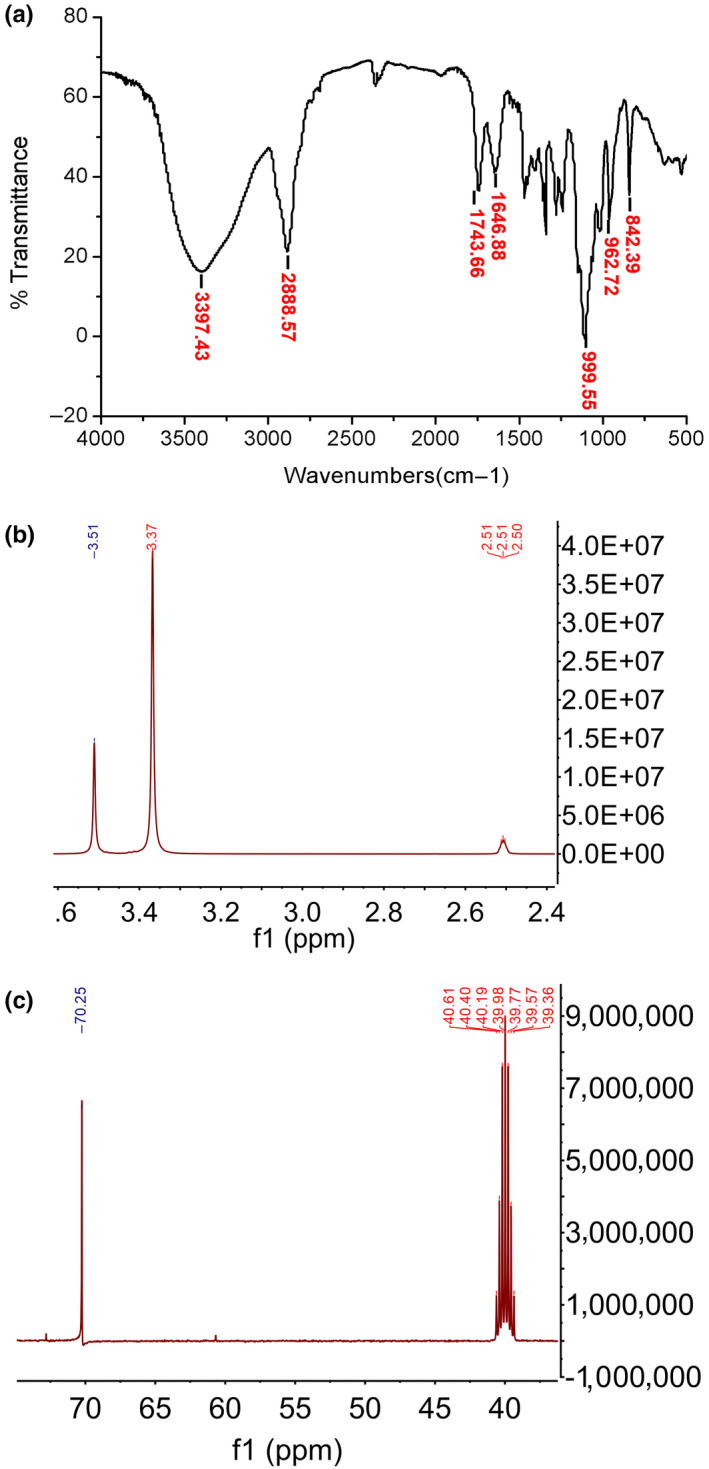
Fourier‐transform infrared spectroscopy (FT‐IR) and nuclear magnetic resonance (NMR) analysis of ASP. (a) FT‐IR analysis of ASP. Significant absorption peaks of ASP occurred in 3,397.43 per cm (getting rise from ‐NH_2_ stretching vibration), 2,888.57 per cm (getting rise from ‐CH_2_ stretching vibration), 1,743.66 per cm (getting rise from β‐NH stretching vibration), and 1,646.88 per cm (getting rise from ‐C‐H‐ bending vibration). Peak signals appeared in 962.72 and 842.39 per cm indicated that ASP possessed α‐ and β‐configuration. (b) H1NMR spectroscopy of ASP. Proton peak signals were crowded in the range of 3.3–3.5 ppm, which was the typical of proton peaks of sugar ring. (c) C13NMR spectroscopy of ASP. Peak signals in C13 NMR were concentrated in 39–40 ppm

As depicted in Figure 2a, the significant absorption peaks of ASP occurred in 3,397.43 per cm (getting rise from ‐NH2 stretching vibration), 2,888.57 per cm (getting rise from ‐CH_2_ stretching vibration), 1,743.66 per cm (getting rise from β‐NH stretching vibration), and 1,646.88 per cm (getting rise from ‐C‐H‐ bending vibration). Additionally, the peak signals appeared in 962.72 and 842.39 per cm indicated that ASP possessed α‐ and β‐configuration. The use of NMR to identify the structure of polysaccharides is a breakthrough progress. H^1^ NMR is applied in determine the conformation of glycosidic bond. As presented in Figure 2b, the proton peak signals are crowded in the range of 3.3–3.5 ppm, which is the typical of proton peaks of sugar ring. In C^13^ NMR spectrum, the absence of peaks in the range of 80–88 ppm revealed that all sugar residues were in the form of pyranose (Figure 2c). Peak signals in C^13^ NMR are concentrated in 39–40 ppm. Results in the NMR analysis are in line with FT‐IR.

### 3.2 ASP alleviates NAFLD in vitro

#### 3.2.1 Cell culture and treatment

**Figure 3:**
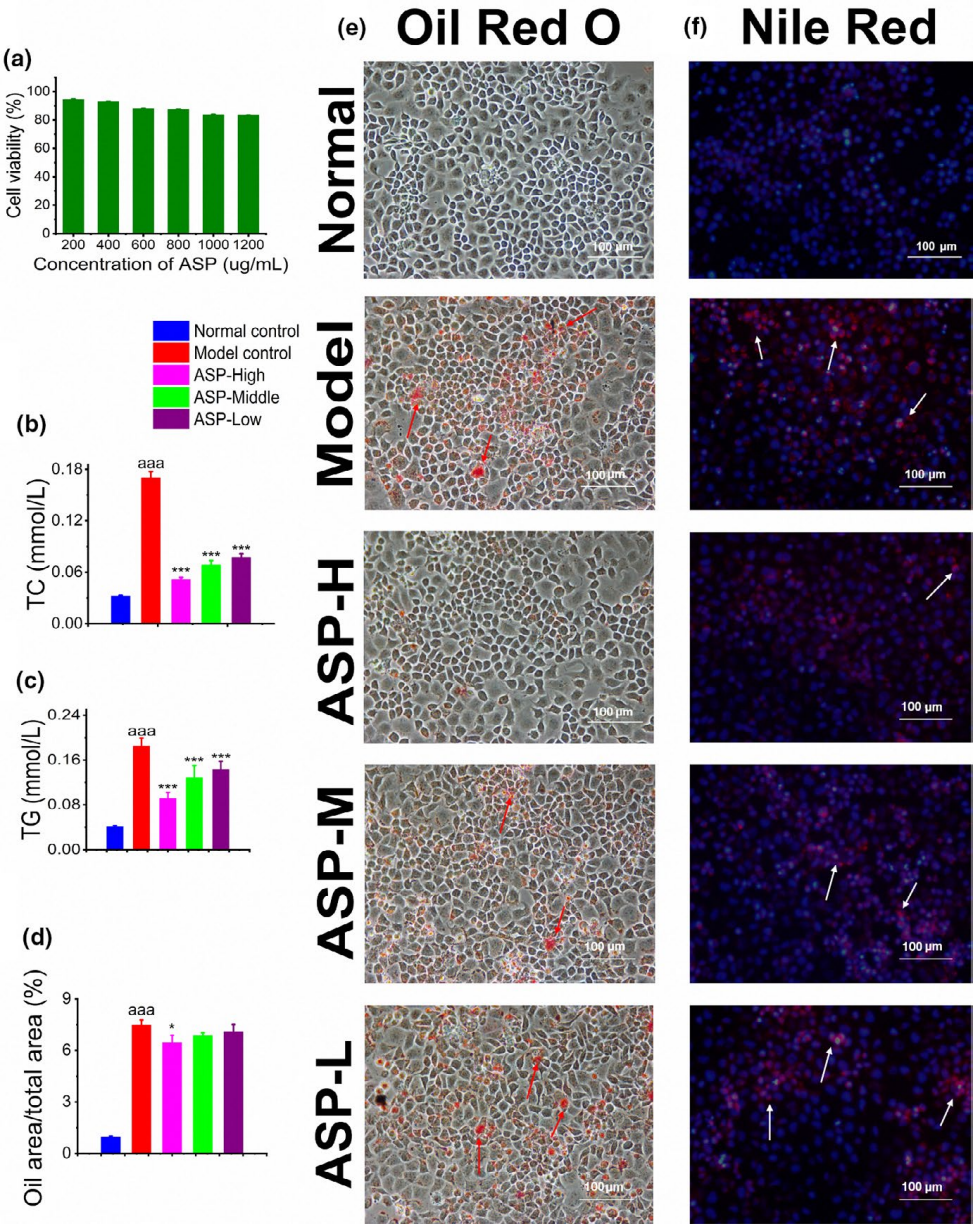
The assessment on NAFLD model in vitro. (a) Cytotoxicity assay. Cell viability in all ASP treatment groups (from 200 to 1,200 μg/ml) was above 80%. (b) The TC level in cells supernatant. TC in model control was significantly higher than normal control group (^aaa^ *p* < .001). All ASP groups were significantly different from model control group (***p < .001) and ASP reduced TC level in a dose‐dependent manner. (c) The TG level of cells supernatant. TG in model control was significantly higher than normal control group (^aaa^ *p* < .001). All ASP groups were significantly different from model control group (***p < .001) and ASP reduced TG level in a dose‐dependent manner. (d) Semiquantitative analysis of Oil Red O staining. Oil Red O (ORO)‐positive area in model control group was significantly higher than normal control group (^aaa^ *p* < .001). The ASP‐high group significantly reduced ORO‐positive area (*p < .05). (e) Oil Red O staining assessment. ASP reduced red lipid in a dose‐dependent manner (scale bar 100 μm). (f) Nile red staining assessment. ASP alleviated red fluorescent intensity in a dose‐dependent manner (scale bar 100 μm)

Results of MTT suggested that ASP in spread range of concentration (from 200 to 1,200 μg/ml) had no cytotoxicity and cell viability was above 80% in all groups (Figure 3a). Thus, the low‐toxicity ASP could be applied in subsequent experiments. The current therapy methods for NAFLD containing compounds, chemical drugs, and dietary supplements are mostly focused on only one target, which are usually insufficient to eliminate the adverse effects caused by a poor diet and a sedentary lifestyle. Therefore, a satisfactory treatment targeting several pathological mechanisms of NAFLD are yet to be unearthed. Numerous studies (Cao et al., 2018; Liu et al., 2019; Wang, Cao, et al., 2016; Zhang et al., 2016; Zhuang, Wang, Zhang, & Xu, 2018) have reported that ASP possessed a variety of health properties, which include anti‐inflammatory, antioxidant, antitumor, and hepatoprotective, which can be considered as potential therapeutic option for NAFLD and intervene in various pathological mechanisms.

Usually, FFA appears to be prominent mediators of lipo‐toxicity which are potential cellular toxins to induce lipid overaccumulation through insulin resistance (IR; Zhang et al., 2014). Insulin resistance is an important underlying cause of NAFLD (Gaggini et al., 2018). Therefore, in vitro NAFLD model was established in L02 cell lines via FFAs induction.

#### 3.2.2 Effect of ASP on intracellular lipid profile

As shown in Figure 3b, the level of TC displayed a significant increase from 0.033 ± 0.001 mmol/L in normal group to 0.17 ± 0.007 mmol/L in model groups, which increased by around 80% and suggested that the NAFLD was successfully induced in the L02 cells (^aaa^ *p* < .001). Notably, TC in ASP‐treated groups (high, middle, and low) decreased to 0.05 ± 0.003, 0.07 ± 0.005, and 0.078 ± 0.004 mmol/L, respectively, which showed significant difference from model control group (^***^ *p* < .001). Figure 3c exhibited the level of TG in cell supernatant. Similarly, data in model group suggested a significant increase (0.18 ± 0.014 mmol/L, ^aaa^ *p* < .001) in comparison with normal cells (0.041 ± 0.002 mmol/L). Moreover, TG level in high, middle, and low dosage of ASP decreased by 50%, 28%, and 22%, respectively, which indicating that ASP had significant effect on alternation of TG accumulation (^***^ *p* < .001). Based on the analysis of these results, ASP could decrease the lipid profile (TC and TG) in a dose‐dependent manner with the high dose of ASP exhibiting the best effects.

#### 3.2.3 Effect of ASP on oleic acid lipid accumulation

Notably, ORO is a fat‐soluble dye extensively used for qualification and quantification of neutral triglycerides and lipids (Koopman, Schaart, & Hesselink, 2001). As shown in Figure 3d–e, the model group with extensive red color indicated inflammatory in the liver cells. As the dosage of ASP increases, the ORO‐positive area also dropped, which suggests a decreased level of lipid accumulation in the hepatocytes. The ASP‐H group could significantly decrease ORO‐positive area (^*^ *p* < .05).

#### 3.2.4 Effect of ASP on neutral lipid accumulation

Nile red is a lipid fluorescent dye capable of labeling neutral lipid pigments and tracing the lipid distribution in the liver (Sheu, Tsai, Lin, Wong, & Lee, 2003). As depicted in Figure 3f, the model group was photographed with a significant red fluorescent intensity indicating that the FFAs‐induced lipid accumulation in L02. Importantly, ASP could attenuate the fluorescence intensity dose‐dependently. Briefly, ASP was capable of ameliorating FFA‐induced NAFLD in vitro through the reduction of lipid profile, lipid droplets, and inflammatory in the liver cells.

### 3.3 ASP alleviates NAFLD in vivo

#### 3.3.1 Acute oral toxicity study in mice

**Figure 4:**
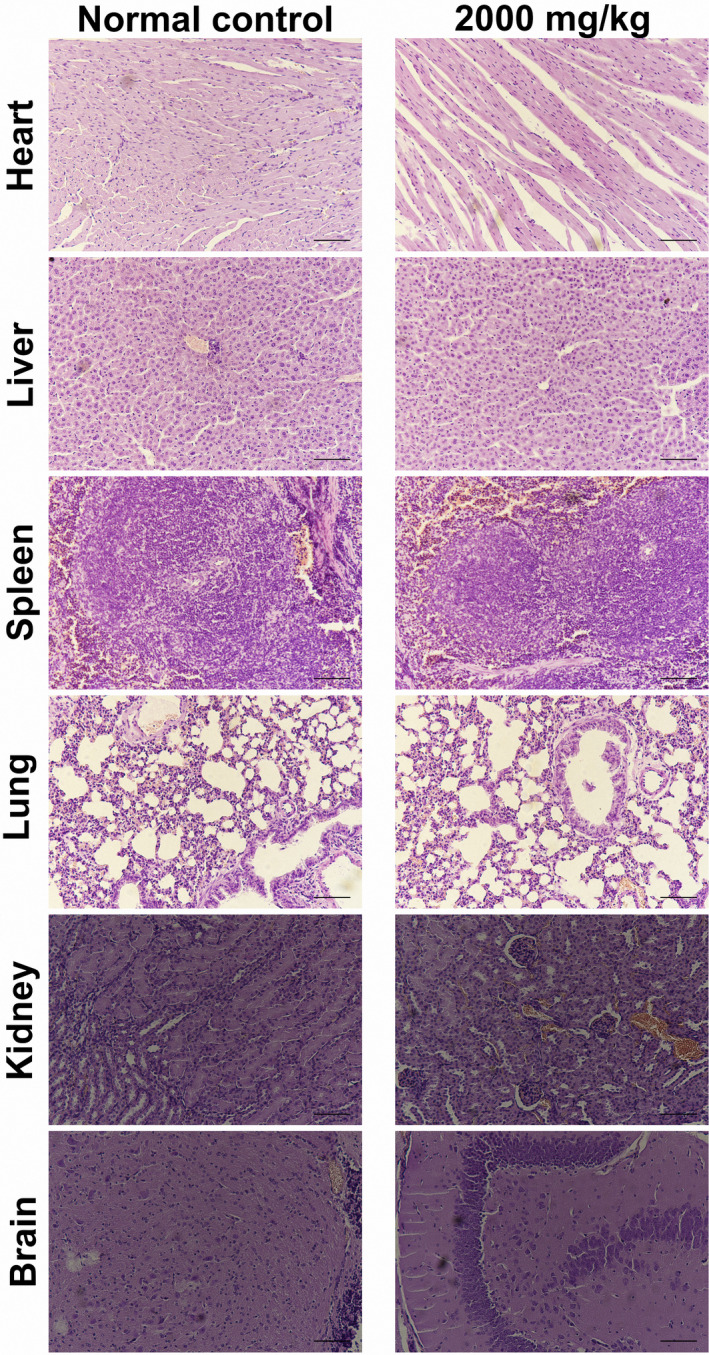
Acute oral toxicity study in mice. ICR mice treated with ASP in a dosage of 2,000 mg/kg body weight shown no obvious variations and injuries on internal organs, including heart, liver, spleen, lung, kidney, and brain (scale bar 50 μm)

During the 2‐week period, mortality of ICR mice was zero. No significant variations were observed in the fur, skin, and eye color. The finding of HE staining (Figure 4) revealed no organic injury in all internal organs, including heart, liver, spleen, lung, kidney, and brain. Thus, LD_50_ value of ASP was greater than 2,000 mg/kg. Numerous studies (Chen et al., 2016; Deore & Mahajan, 2018) reported that LD_50_ value of natural polysaccharides was greater than 2 g/kg and could be designated as practically nontoxicity. Therefore, ASP was relatively safe and no side effects.

#### 3.3.2 Animals and experimental designs

Mice fed with HFD for a consecutive long time could exhibit typical symptoms of NAFLD viz., ectopic lipid accumulation, fatty regeneration, and abnormality of serum lipid profiles, which is caused by a faster synthetic rate of TG and cholesterol than their transporting rates (Nakamura & Terauchi, 2013). The model of NAFLD was established in mice after feeding them with HFD for 45 days. The body weights of the model and normal groups were recorded every three days, and the body weight in the model group was increased by twofold while the normal group reached 30 g and maintained in this level from Day 20 (data not shown). After modeling, the ASP groups (100, 300, and 500 mg/kg) were intragastrically fed on a daily basis for 10 consecutive days while the normal group was given a regular animal chow.

#### 3.3.3 Effect of ASP on serum lipid profile

**Figure 5:**
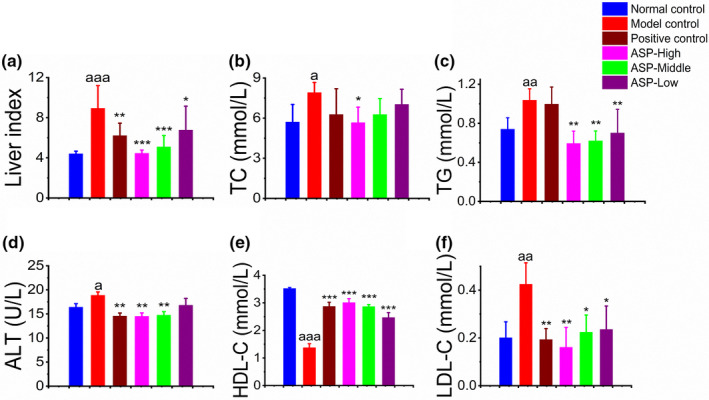
Effects of ASP on serum lipid profile in vivo. (a) The liver index. Liver index in model control was significantly higher than normal control group (^aaa^ *p* < .001). Liver index in ASP‐high and ASP‐middle groups significantly decreases (***p < .001). (b) The level of serum TC. TC in model control group was higher than in normal group (^a^ *p* < .05). TC in ASP‐high group was significantly lower than model control group (*p < .05) and ASP decreased serum TC in concentration‐dependent manner. (c) The level of serum TG. TG in model control group was higher than in normal group (^aa^ *p* < .01). TG in ASP‐treated group was significantly lower than model control group (**p < .01) and ASP decreased serum TG in concentration‐dependent manner. (d) The level of serum ALT. ALT decreased with an increase in the concentration of ASP. ASP‐high group and ASP‐middle group were significantly different from model control group (**p < .01). (e) The level of serum HDL‐C. HDL‐C decreased with an increase in the concentration of ASP. ASP‐treated groups were significantly different from model control group (***p < .001). (f) The level of serum LDL‐C. The level of LDL‐C decreased with an increase in dosage of ASP. LDL‐C level in ASP‐high group was decreased significantly (**p < .01)

After sacrificing the mice, liver index and serum lipid parameters of the mice were examined. Compared with normal control, liver weight in model control group significantly increased due to hepatic lipid accumulation and development of fatty liver (^aaa^ *p* < .001; Figure 5a). Liver index in all the ASP groups was less than the model group indicating that ASP could attenuate liver swelling in NAFLD. Additionally, the ASP could decrease liver index in a dose‐dependent manner. The serum lipid profile was a critical indicator which reflects the liver damage state. For example, ALT and AST are well‐known diagnostic indicators of liver disease and the upgoing of ALT and AST indicated the injury of liver tissues. As shown in Figure 5d, ALT level increased from 164.2 ± 7.07 mmol/L in normal group to 188.6 ± 7.07 mmol/L in model control, indicating the success of NAFLD modeling in ICR mice (^a^ *p* < .05). Meanwhile, mice treated with ASP in 300 and 500 mg/kg showed a significant decrease of ALT. The values of these two groups are 145.09 ± 7.07 mmol/L (**p < .01) and 147.53 ± 7.07 mmol/l (**p < .01), respectively. An increase in plasma LDL‐C, TG, and TC levels and a decrease in circulating HDL‐C level were recognized as major risk biomarkers for NAFLD. As displayed in Figure 5b, TC in model control is 7.9 ± 0.76 mmol/L, which is significantly higher than normal group (5.7 ± 1.31 mmol/L, ^a^ *p* < .05). Mice treated with high dosage of ASP (500 mg/kg) displayed a significant reduce in TC level (5.65 ± 1.16 mmol/L, *p < .05) while the other two ASP groups showed a slight decrease with no distinct significance. As shown in Figure 5c, TG in mice treated with ASP in dose of 100, 300, and 500 mg/kg reduced by 43.2%, 40.4%, and 32.7%, respectively, which illustrating a significant decrease compared with HFD‐induced mice (**p < .01). Serum HDL‐C level is 3.01 ± 0.14, 2.87 ± 0.07, and 2.47 ± 0.18 mmol/L, respectively, in the ASP‐high, ASP‐middle and ASP‐low groups (Figure 5e). HDL‐C in all these ASP groups was significantly higher than HDL‐C in model control (***p < .001). The alternation of serum LDL‐C profile (Figure 5f) was similar with HDL‐C, and all ASP‐treated groups were significantly different with model control, which, respectively, decreased by 62.9%, 47.6%, and 45.2% in comparison with model control (***p < .001). Collectively, these findings indicate that lipid‐lowering effect of ASP was manifested in a dose‐dependent manner.

#### 3.3.4 Effect of ASP on histopathology status of HFD‐injured liver

**Figure 6:**
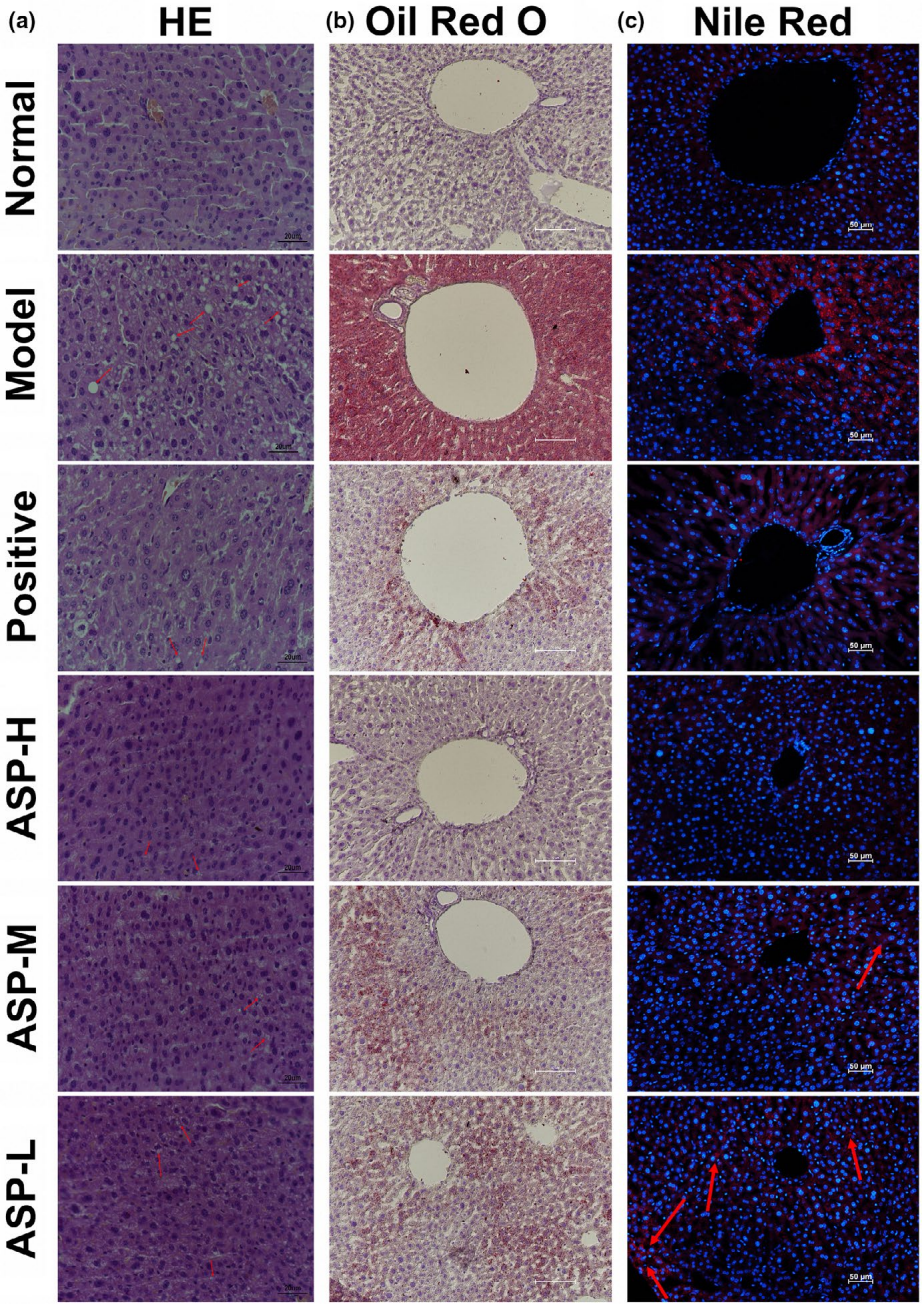
Effect of ASP on histopathology status of HFD‐injured liver. (a) HE assessment. With increasing dosage of ASP, ballooning lesions and macro‐vesicular steatosis scale decreased (scale bar 20 μm). (b) Oil Red O staining assessment in vivo. ASP reduced red lipid in a dose‐dependent manner (scale bar 10 μm). (c) Nile red staining assessment in vivo. ASP alleviated red fluorescent intensity in a dose‐dependent manner (scale bar 50 μm)

As shown in Figure 6a, HE liver tissue sections in the various groups displayed different degrees of fatty degeneration. In the normal groups, distinct hepatic cells with abundant cytoplasm and prominent nucleus could be observed. Moreover, hepatic cords radiated in regular rays from central veins in all directions. However, liver tissue treated with HFD exhibited extensive ballooning lesions and macro‐vesicular steatosis scale which typically demonstrated NAFLD characteristics. With increasing dosage of ASP, hepatocytes ballooning decreased indicating an effective treatment for liver lipid degeneration. The results of ORO (Table 2; Figure 6b) and Nile red staining (Figure 6c) were agreement with the HE findings. ORO‐positive area in ASP‐high group and ASP‐middle group reduced significantly compared model control group (***p < .001). The reducing fluorescence intensity in ORO staining suggested the downregulating of oleic acid lipid droplets while the decreasing fluorescence intensity in Nile red staining indicated the downregulating of neutral lipid deposition. Findings in histological assessment were directly verified ASP intensive effects on alternation of serum lipid profiles and modification of lipid metabolism. In short, ASP could be designated as a therapeutic option for NAFLD treatment in vivo via lowering of plasma lipid profiles, ectopic lipid accumulation, and fatty degeneration.

**Table 2:** Semiquantitative of Oil Red O staining (*n* = 3, mean ± *SD*)

Group	Oil area/total area (%)
Normal control	4.01 ± 0.30
Model control	7.40 ± 0.28^aaa^
Positive control	5.02 ± 0.26***
ASP‐high	4.83 ± 0.56***
ASP‐middle	5.54 ± 0.14***
ASP‐low	6.94 ± 0.13

^aaa^ *p* < .001, compared to normal control group.

*** *p* < .001, compared to model control group.

Finally, it was concluded that ASP with a relatively low MW purified via easy‐repeated “one‐step” process has remarkable advantages such as high stability and convenient availability which may facilitate its clinical application. Additionally, oral administration of ASP with low MW could ameliorate hepatic lipid accumulation and prevent the risk of extensive liver deterioration.

## 4 CONCLUSION

In the present study, we firstly obtained a novel polysaccharide with low MW from dried roots of *A. sinensis* through an optimized, easy‐repeated “one‐step” purification technology. In vitro and in vivo evaluation showed that “small” ASP exhibits its lipid‐lowering effects on NAFLD via possible involvement in lipid metabolism. Thus, ASP could reduce serum and supernatant lipid profiles, lipid pigments deposition, and fatty degeneration, which suggest the prospect of “small” ASP as an effective therapeutic option for NAFLD. Nevertheless, the exact mechanistic action of “small” ASP on NAFLD and the other bioactivities of “small” ASP are an on‐going research in our laboratory.

## CONFLICT OF INTEREST

The authors declared that there is no conflict of interest.

## AUTHOR CONTRIBUTION

Ping Ma was responsible for operating the experiments and writing the manuscript. Congyong Sun helped for designing the experiments and doing experiments. Wenjing Li helped collating experimental data and doing experiments. Wenwen Deng assisted the experimental operation and data processing. Michael Adu‐Frimpong helped revising the manuscript. Ximing Xu and Jiangnan Yu (corresponding author) conducted the experimental design and revised the manuscript.

## ETHICAL STATEMENTS

The experimental protocol was approved by the Institution of Animal Ethical Committee, and the study was carried out in accordance with the National Institute of Health Guide for Care and Use of Laboratory Animals.
